# Histidine Residues at the Active Site of the *Pasteurella multocida* Toxin

**DOI:** 10.2174/1874091X00701010007

**Published:** 2007-06-15

**Authors:** Gillian D Pullinger, Alistair J Lax

**Affiliations:** King’s College London, Dental Institute, Department of Microbiology, London SE1 9RT, UK

**Keywords:** *Pasteurella multocida*, toxin, PMT, domain structure, histidine residues, G-proteins

## Abstract

We have investigated histidine residues near the active site of the mitogenic *Pasteurella multocida* toxin. Mutation of H1202 or H1228 had little effect, while the effect of mutation on H1223 depended on the amino acid substituted. Mutation of H1205 caused complete loss of activity, indicating its importance in PMT activity.

## INTRODUCTION

The *Pasteurella multocida* toxin (PMT) is a potent mitogen for fibroblasts [1]. PMT also affects bone cells, acting as a mitogen for osteoblasts and stimulating bone resorption *in vitro* [2–5]. PMT stimulates members of the G_q_ and G_12_ families of heterotrimeric G-proteins [6–9], and these signalling proteins are thought likely to be the molecular targets of PMT. Their activation by PMT leads to numerous downstream sequelae including activation of phospholipase Cβ [6,10] and Rho-linked signalling [11,12].

PMT is the only toxin known to activate members of the G_q_ and G_12_ G-protein families and is thus likely to become a useful tool for studying pathways linked to these important signalling molecules. We have already used PMT to investigate the function of tyrosine phosphorylation in the activation of G_q_ [8] and to identify the role of the Rho GTPase in osteoblast function [5].

PMT is an intracellularly-acting toxin of 1285 amino acids (146 kDa), which comprises a receptor binding domain at the N-terminus, followed by a translocation domain, and an active domain at the C-terminus [13–15]. We have previously analysed the role of cysteine residues in the toxin molecule and identified an essential role for C1165 [16]. Mutation of this residue completely abrogated activity without grossly affecting the toxin structure. Orth and co-workers subsequently suggested that 2 histidine residues within the same region (H1205 and H1223) were essential for activity [17]. Recently, Kitadokoro and co-workers have crystallised the C-terminal domain and suggest that it comprises 3 subdomains, including a domain (the C3 domain) that contains a catalytic triad that consists of C1165, H1205 and D1220 (PDB identifier 2EBF) [18]. We have analysed the role of all 4 histidine residues in the C3 subdomain and show here that H1205 is critical for activity, whereas mutation of H1223 has only a partial effect that is dependent on which amino acid replaces H1223.

## MATERIALS AND METHODS

### Materials

All chemical reagents were from Sigma, Ltd., unless otherwise stated. Oligonucleotides were synthesised by Sigma Genosys. Restriction endonucleases and other enzymes were purchased from Promega Corporation or New England Bio-labs. Chromatography reagents and radioisotopes were purchased from Amersham Bioscience unless stated otherwise.

### Bacterial Strains

*Escherichia coli* XL1-Blue was the host for pTox2, which expresses full-length recombinant PMT (UniProt code http://www.expasy.org/sprot/userman.html - AC_line P17452) from its own promoter [16], and for pTox2 C-terminal deletants and point mutants. *E. coli* strains were routinely cultured in Luria-Bertani (LB) broth or on LB agar aerobically (at 30 ^°^C for pGEX constructs or at 37 ^°^C for pTox2 and its derivatives). Antibiotics were added as appropriate at the following concentrations; tetracycline, 20 μg ml^-1^; and ampicillin, 100 μg ml^-1^.

### DNA Sequencing, Site Directed Mutagenesis

Plasmid DNA was isolated by using Wizard kits (Promega Corporation). For sequence analysis, DNA was further purified by using Qiaquick columns (Qiagen). DNA sequencing was performed with a Beckman CEQ2000 automated DNA sequencer. PMT histidine residues 1202, 1205, 1223 and 1228 were replaced individually with leucine and with tyrosine using the Stratagene Quikchange system. Plasmid pTox2 [16], which encodes the native toxin, was used as the template for site-directed mutagenesis reactions using oligonucleotide primers of 25 nucleotides length designed to generate the desired mutations. Two separate colonies of each of the eight potential mutants were stored for further analysis.

### Purification and SDS-PAGE Analysis of Recombinant Proteins

PMT and untagged toxin derivatives were purified as previously described from cleared lysates by anion exchange chromatography followed by hydrophobic interaction chromatography [16]. Glycerol was added to 50 % (v/v), and preparations were stored at -20 ^°^C for up to 6 months. Proteins were separated in denatured form on 4 % stacking and 8 % resolving gels [19]. Proteins were visualised by a silver staining technique as described previously [20].

### Assays for PMT Activity

Swiss 3T3 fibroblasts were maintained as described in reference 1. DNA synthesis was measured by the incorporation of [^3^H]thymidine into quiescent Swiss 3T3 cells as described [21]. The cell-binding of PMT fragments was assessed by determining their ability to compete with PMT in DNA synthesis assays as described previously [13]. For actin staining, cells were fixed in 3.7 % (w/v) formaldehyde in PBS for at least 10 min. This was followed by permeabilisa-tion for 5 min in the presence of 0.5 % (v/v) Triton X-100/PBS and blocking for 10 min with 1 % (w/v) BSA, 100 mM glycine. Cells were then placed in 0.5 μg TRITC-phalloidin ml^-1^ for 30 min (each step was preceded by 3 washes in PBS). Stained cells were washed and mounted in Vectashield (Vector Laboratories). Total inositol phosphates were assayed as described [22]. Briefly, [^3^H]*myo*-inositol-labeled Swiss 3T3 cells were pretreated with 17.5 pM PMT or mutants for 4.5 h. Inositol phosphates were prepared and expressed as cpm/well

### Proteolysis

The susceptibility of PMT and its mutants to proteolysis by the serine protease Glu-C was assayed in the presence of different concentrations of SDS as previously described [23].

## RESULTS

**Fig. (1):**
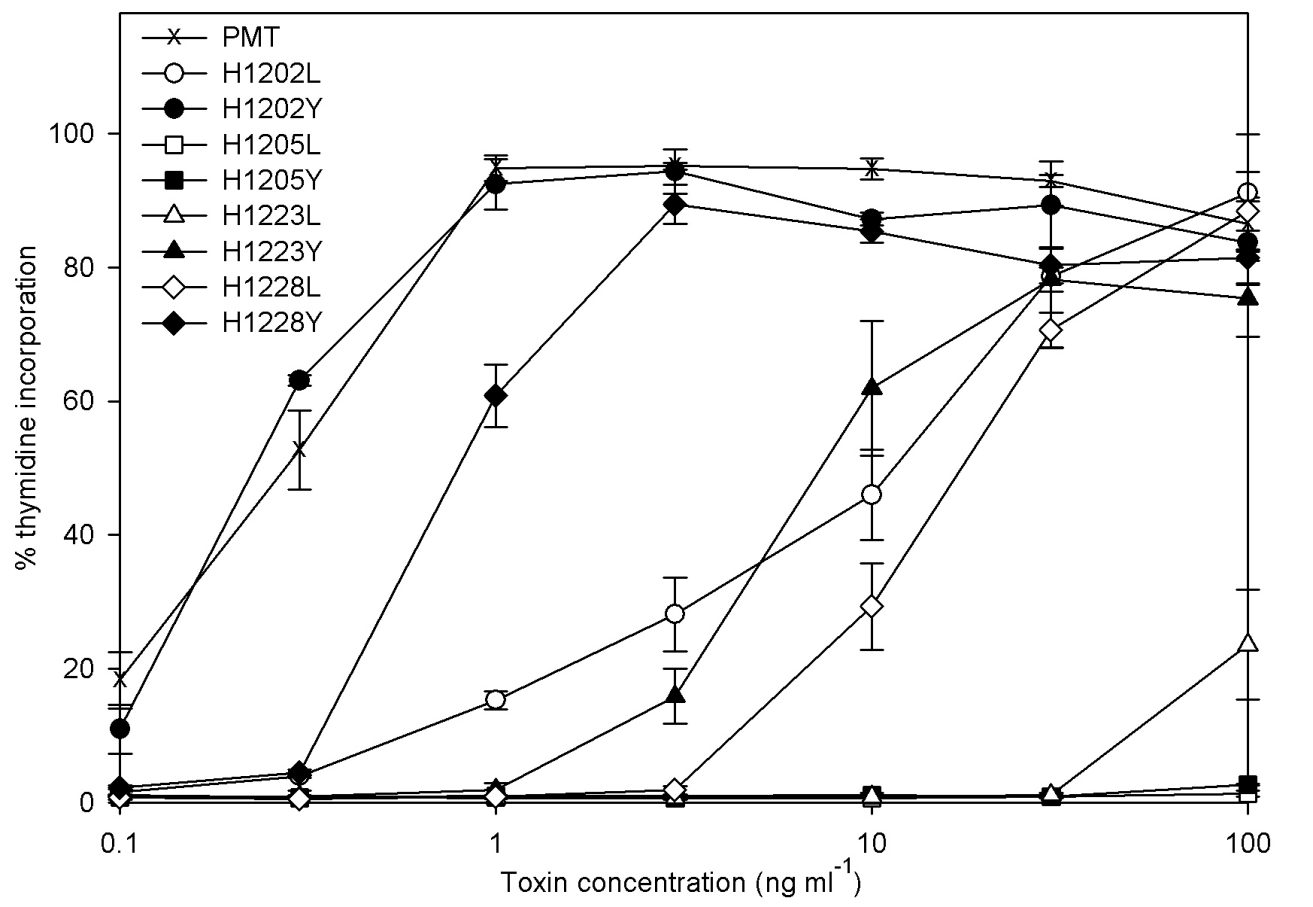
Induction of DNA synthesis in quiescent Swiss 3T3 fibroblasts by PMT and mutants. The results are expressed as a percentage of the counts obtained following stimulation with 10 % foetal bovine serum. The results presented are the means and standard errors from six determinations at each point

The four published sequences of the PMT gene show a high degree of conservation and the four histidine residues analysed here are conserved and in the same context in each sequence. Histidine point mutants of PMT (PMT^H1202L^, PMT^H1205L^, PMT^H1223L^, PMT^H1228L^, PMT^H1202Y^, PMT^H1205Y^, PMT^H1223Y^ and PMT^H1228Y^) were prepared as described in Methods. Sequencing confirmed that at least one isolate of each had the desired mutation. The 8 mutant toxins were all expressed in similar amounts to wild-type PMT (not shown). The relative activities of these mutant toxins were assessed in a number of assays. The ability of purified toxins to induce DNA synthesis in quiescent Swiss 3T3 cells was compared with normal PMT. PMT^H1205L^ and PMT^H1205Y^ were completely inactive up to a concentration of 100 ng ml^-1^, whilst wild-type toxin stimulated DNA synthesis at as little as 0.3 ng ml^-1^ (Fig. **1**). PMT^H1202Y^ was as active as wild type toxin, whereas PMT^H1228Y^ was attenuated about 3-fold. Interestingly, PMT^H1223Y^ was around 30 times less active than wild-type toxin. For each of the 3 pairs of mutations, the associated leucine mutants were attenuated around a further 30-50 fold compared to the tyrosine substitutions. This meant that PMT^H1223L^ was at least 1000-fold less active than wild-type PMT

**Fig. (2):**
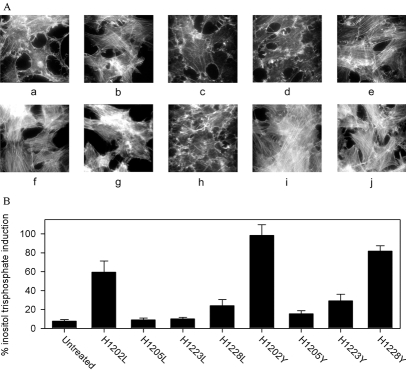
Effects of histidine mutants of PMT. A On the formation of actin stress fibres in quiescent Swiss 3T3 cells. Toxins were added at 5 ng ml^-1^ for 16 h. Additions were as follows: (**a**), untreated; (**b**) PMT^H1202L^; (**c**) PMT^H1205L^; (**d**) PMT^H1223L^; (**e**) PMT^H1228L^; (**f**) PMT(**g**) PMT^H1202Y^; (**h**) PMT^H1205Y^; (**i**) PMT^H1223Y^; (**j**) PMT^H1228Y^. B On the induction of inositol trisphosphates. Inositol phosphates were assayed in quiescent Swiss 3T3 cells that had been labelled with ^3^H-inositol for 20 h, then treated for 4h with 20 ng ml^-1^ mutant toxins or left untreated. The results were expressed as a percentage of the counts obtained for wild-type PMT. The bars represent the mean and standard errors for six determinations at each point

The effect of the amino acid substitutions on the ability to induce cytoskeletal rearrangements was determined. PMT induced the formation of thick, parallel stress fibres, whereas untreated cells contained disorganised actin fibres (Fig. **2A**). Only the three histidine mutants that demonstrated the least activity in the thymidine incorporation assay, PMT^H1205L^, PMT^H1205Y^ and PMT^H1223L^, failed to induce actin stress fibre formation under these conditions. The effect of the mutations on the activation of G_q_ was tested by assessing the ability of the mutant toxins to stimulate production of inositol trisphosphate (IP_3_). PMT^H1205L^, PMT^H1205Y^ and PMT^H1223L^ did not significantly induce inositol phosphate formation (Fig.**2B**), showing that they failed to activate G_q_. The other mutants were all partially active. These results are summarised in Table 1.

**Fig. (3):**
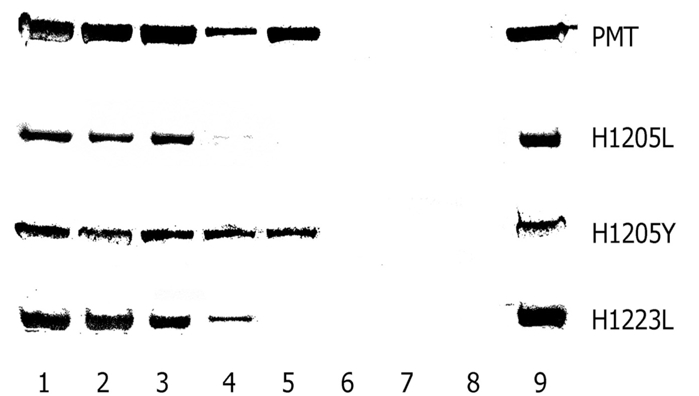
Resistance of PMT and selected histidine mutants to digestion with the protease, Glu-C. One microgram of PMT or mutant toxins was incubated with 25 ng Glu-C (Sigma) in the presence of different concentrations of SDS for 1 h at 37 ^°^C. The SDS concentrations used were: lane 1, no SDS; 2, 0.005 %; 3, 0.075 %; 4, 0.01 %; 5, 0.0125 %; 6, 0.015 %; 7, 0.02 %; 8, 0.025 %. Lane 9 contains toxin with no Glu-C. The products were electrophoresed through 10 % SDS-PAGE gels, and detected by silver staining. The toxin bands of 146 kDa are shown for PMT, PMT^H1205L^, PMT^H1205Y^ and PMT^H1223L^

The three mutants with a marked reduction in activity were tested for proteolytic resistance to the protease Glu-C to determine whether these mutations had introduced gross structural changes that affected protein stability. We have previously shown that this technique can identify subtle differences in structure that are undetectable by circular dichroism [16]. The susceptibility of these mutant toxins was tested at a range of SDS concentrations as previously described [23]. PMT^H1205Y^ became susceptible to proteolytic cleavage at the same SDS concentration as PMT (Fig.**3**), suggesting that it was structurally unaltered. PMT^H1205L^ and PMT^H1223L^ were a little more sensitive to SDS denaturation, suggesting that they possessed slightly altered structures.

**Fig. (4):**
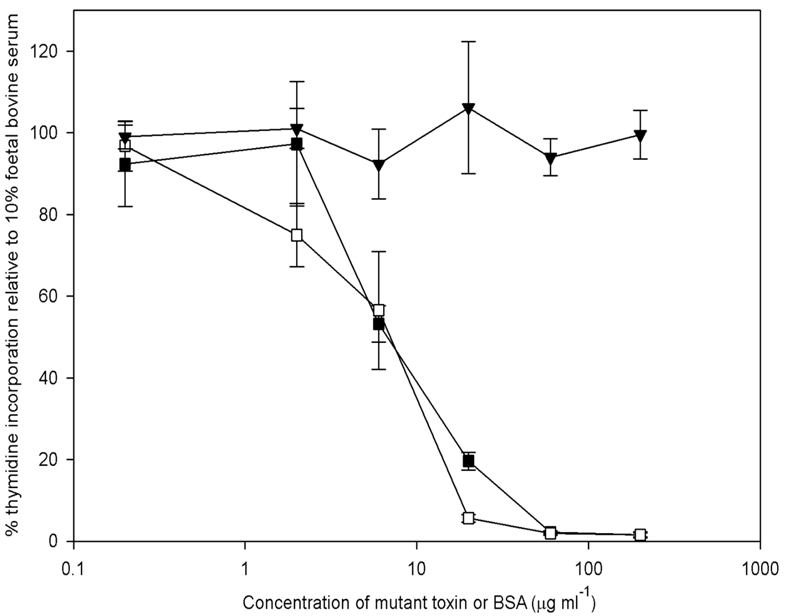
Blocking of PMT-induced DNA synthesis by PMT^H1205L^ and PMT^H1205Y^. DNA synthesis was assayed in quiescent Swiss 3T3 cells with PMT at 2ng ml^-1^ in the presence of an excess of the mutant toxins. Results are expressed as the percentage of counts obtained with 10 % foetal bovine serum. BSA was used as a control. The blocking proteins were: (▪) PMT^H1205L^; (□) PMT^H1205Y^; (▾) BSA. The results presented are the means and standard errors from six determinations at each point.

The inactive mutants PMT^H1205L^ and PMT^H1205Y^ were tested for their ability to bind to cells, using the competition assay to assess whether they could block the mitogenic activity of 2 ng PMT ml^-1^. Each mutant significantly inhibited the mitogenic action of PMT when added to a concentration of 6 μg ml^-1^ or higher (Fig. **4**).

## DISCUSSION

A critical role for histidine residues in PMT activity was first suggested by Orth and co-workers [17], who proposed that H1205 and H1223 were essential for PMT function. Kitadokoro and colleagues have recently suggested that C1165, H1205 and D1220 form a catalytic triad that might facilitate either proteolytic or acyltransferase activity [18], while H1223 faces away from the putative active site.

We have analysed the role of all the histidine residues within the catalytic C3 domain and have shown here that H1223 does not have as critical a role in activity as H1205. Substitution of H1205 with either tyrosine or leucine leads to complete loss of activity, without affecting cell binding activity. In addition, the PMT^H1205Y^ substitution mutant shows little evidence of gross structural changes. We confirm that PMT^H1223L^ shows almost no activity in the most sensitive test of activity, the ability to induce thymidine incorporation, while the PMT^H1223Y^ mutant retained substantial but reduced activity (around 3% of the activity of wild-type toxin). Mutation of the two other histidine residues in this region had little or no effect if tyrosine was substituted, although a substantially greater effect when replaced by leucine. This is probably due to the relatively conservative nature of the histidine to tyrosine mutation, as both residues are polar and, aromatic. In contrast leucine is non-polar and smaller so is more likely to affect the structure of the toxin. We have shown here that leucine substitution leads to greater structural instability, as assessed by protease sensitivity. Similarly, PMT^C1165S^ is more protease sensitive [16]. Structural analysis of the catalytic domain of PMT^C1165S^ has confirmed that it has a slightly altered structure, which correlates with the observed increase in protease sensitivity [18]

## CONCLUSION

Taken together, H1202 and H1228 appear to play no direct role in the PMT catalytic centre. Mutation of H1223 has a greater effect on activity, presumably because of its structural proximity to the catalytic centre, but our work supports the suggestion by Kitadokoro that H1223 is unlikely to be directly involved in catalysis [18]. The demonstration that mutation of H1205 has a major effect on PMT activity supports the concept that C1165, H1205 and D1220 are essential for catalysis. The identification of critical amino acid residues in the catalytic domain and the further definition of that domain should aid future studies on its function

## Figures and Tables

**Table 1.** Relative Activity of PMT Mutants

Mutant	Mitogenicity	Inositol trisphosphate induction	Stress fibre formation
H1202L	57	56	+
H1205L	1	1	-
H1223L	10	3	-
H1228L	45	18	+
H1202Y	96	98	++
H1205Y	1	8	-
H1223Y	52	23	+++
H1228Y	83	80	+++

Summary of data in Figs. **1** and **2**. The values given for mitogenicity are the average of induced thymidine incorporation relative to that induced by 10% foetal calf serum at 1, 10 and 100ngml^-1^ PMT (Fig. **1**). Inositol trisphosphate induction is the induction above the untreated value relative to that induced by 10% foetal calf serum (Fig. **2**). The induction of stress fibres formation is scored on the basis of-no induction;+evidence of stress fibre formation; ++ between+and +++; +++ stress fibre induction equivalent to that induced by PMT (Fig. **2**)
